# Recovery rate and its predictors among children with severe acute malnutrition in Addis Ababa, Ethiopia: A retrospective cohort study

**DOI:** 10.1371/journal.pone.0235259

**Published:** 2020-07-23

**Authors:** Zebenay Workneh Bitew, Animut Alebel, Teshager Worku, Ayinalem Alemu

**Affiliations:** 1 Department of Pediatric Nursing, School of Nursing, St. Paul’s Hospital Millennium Medical College, Addis Ababa, Ethiopia; 2 College of Health Science, Debre Markos University, Debre Markos, Ethiopia; 3 School of Nursing and Midwifery, College of Health and Medical Sciences, Haramaya University, Harar, Ethiopia; 4 Department of Medical Microbiology, Ethipian Public Health Institute, Addis Ababa, Ethiopia; King's College London, UNITED KINGDOM

## Abstract

**Introduction:**

Malnutrition is a public health problem in under-five children in several parts of the world even after decades of the implementation of management protocols. An estimated 17 million children under the age of five years are living with severe acute malnutrition and the majorities are found in Asia and Africa, including Ethiopia.

**Objective:**

The main objective of this study was to determine the recovery rate and its predictors among under-five children who were admitted to St. Paul’s Hospital Millennium Medical College from 2012 to 2019.

**Methods:**

An institution based retrospective cohort study was employed at St. Paul’s Hospital Millennium Medical College from May 20, 2019 to June 28, 2019. Data were collected by reviewing children’s’ medical records using a structured checklist. A total of 534 charts were selected using a simple random sampling method and 515 of them were used for the final analysis. Ep-info version 7 software was used for data entry and STATA Version 15 for analysis. The Kaplan Meier failure estimate with Log-rank test was used to determine the survival estimates. Bi-variable and multivariable Cox proportional hazards regression model were fitted to identify predictors of mortality. Finally, variables with p-values less than 0.05 in the multivariable Cox regression were considered as independent predictors. The proportional hazards assumption was checked using the Schoenfeld residuals test and the final model fitness was checked using the Cox-Snail residual test.

**Result:**

In this study, a total of 515 subjects were followed for 8672 child–days and 79% of the subjects recovered from SAM with the median time of 17 days. The incidence density rate of recovery was 46 per 1000 child-days. Tuberculosis (AHR(Adjusted Hazard Ratio) 0.44 & 95% CI: 0.32, 0.62), pale conjunctiva (AHR,0.67 & 95% CI: 0.52, 0.88), IV fluid infusion (AHR, 0.71 & 95 CI: 0.51, 0.98), feeding F100 (AHR, 1.63 & 95% CI:1.04,2.54), Vitamin A supplementation (AHR, 1.3 & 95% CI:1.07, 1.59) and bottle feeding (AHR, 0.79 & 95CI%: 0.64–0.98) were the independent predictors of time to recovery from SAM.

**Conclusion:**

In conclusion, the recovery rate was relatively higher than the Sphere standard and the national SAM management protocol. Co-morbidities and the treatments given were the main determinants of recovery of children. Co-morbidities must be managed as early as possible and the treatments given during the SAM management process need to be given with precaution.

## Introduction

Malnutrition includes all forms of imbalanced nutrition and severe acute malnutrition (SAM)is the form of under-nutrition commonly affecting children under-five [1]. Severe acute malnutrition is defined as very low weight for height/length (<-3 z score of the median World Health Organization (WHO) growth standard, presence of bilateral edema or mid upper arm circumference (MUAC) < 115 mm for a child ≥ 6 months of age [2].

Malnutrition is a public health problem in children under-five in several parts of the world [3]. In 2018, an estimated 149 million (21.9%), 49 million (7.3%), and 40 million (5.3%) under-five children were living with stunting, wasting and overweight, respectively and the majority (73%) of wasted children were found in low and middle income countries (LMICs) [4]. The growth of one out of every three children under-five is mitigated by imbalanced macronutrient levels and one out of every two children are suffering from hidden hunger which affirms the transition of malnutrition from double to triple burden [5]. An estimated 17 million children were living with SAM in by the 2016 worldwide, of which the majorities (98%) were from Asian and African countries, including Ethiopia [6, 7].

Severe acute severe is a major public health problem of children in Ethiopia where seven percent of under-five children are affected by wasting and 1% of them are severely wasted. There is regional variation in the national distribution of SAM and the highest (21%) and lowest (2%) percentage of wasted children are found in Somalia region and Addis Ababa, respectively [8]. SAM is also an economic burden of the country which increases the direct and indirect costs that contributed to 20% of pediatric hospital admissions [9, 10].

Ethiopia is implementing the national SAM management protocol and Seqota declaration as part of the Second National Nutrition Program (NNP-II), but the recovery rate from SAM is still far from the expected [11]. The previous studies performed in Ethiopia also revealed that the recovery rate from SAM is inconsistent ranging from 43.59% [12] to 87% [13] and most studies [14–21] pinpointed that the recovery rates are lower than the recommended WHO and national Sphere standard of minimum performance indicators, benchmarks against which to interpret the quality and effectiveness of functioning therapeutic feeding programs [22]. These findings affirmed that the cure rate of children with SAM who received treatment at the stabilization centers of the country remained low even after the implantation of the standard treatment guidelines. Since 2009, Ethiopia has adopted and implemented the national and international commitments to end all forms of malnutrition, mainly in children under-five [11]. Nonetheless, SAM continues to affect under-five children in devastating ways. This could be attributed to factors such as clinical, socio-demographic, economical and factors related to inappropriate implementation of the treatment protocols [15–17, 20, 23–28]. However, little is known about the recovery rate and factors associated with time to recovery in the study area. Therefore, this study aimed to determine the recovery rate and its predictors among children admitted with SAM in St. Paul’s Hospital Millennium Medical College (SPHMMC) from 2012 to 2019.

## Methods

### Study area and design

This study was a retrospective cohort study conducted in SPHMMC. Data were collected from May 20, 2019 to June 28, 2019. St. Paul’s Hospital Millennium Medical College was established at the center of the country, Addis Ababa, through a decree of the Council of Ministers in 2010. It has more than 700 beds and an average of 1200 emergency and outpatient clients are seen daily. Children referred from the four corners of the country received therapeutic service for SAM after they are admitted the stabilization centers (SCs) of the hospital. An average of 300 children with SAM is treated per annum in the pediatric ward. One room with an average capacity of 10 beds is reserved as SCs of children with SAM. However, when the caseload is increased, additional rooms can be used as SC. Both physicians and nurses manage children with SAM based on the national nutrition protocol [22].

### Population

All records of under-five children who were admitted to the SCs of SPHMMC from November, 2012 to June 2019 were the source population of this study. A total of 1110 child records were eligible from which 534 were selected by simple random sampling methods using the SPSS version 25 software. All records of under-five children with SAM admitted to SCs were included, but children with incomplete records, unknown admission dates and unknown discharge dates were excluded.

### Sample size determination and sampling procedure

The sample size was calculated using STATA (version 15) through the following statistical assumptions: two-sided significant level (α) of 5%, power 80%, Za/2 = Z value at 95% confidence interval = 1.96, death rate = 69.2%, hazard ratio (HR) = 1.36 [17]. The HRs were taken from the study where the maximum recovery rate was reported and the predictor which gave the maximum sample size (HR = 1.36), was used to calculate the final sample size. Sample size was calculated for Cox proportional hazards model.

By considering all of the independent predictors of recovery from the reference study and by using power and sample size from STATA (version 15) software for Cox proportional hazards model, all the possible sample sizes were calculated and the largest sample size (N = 534) was selected. Simple random sampling technique was implemented to select the participants and the medical registration numbers (MRN) of clients during admission were used to generate 534 random numbers. The SPSS version 25 software was used to generate computer based random numbers. Out of the set of children available (1110), 534 were chosen, of which 515 were had complete information and were used for the final analysis. Finally, the records were collected from the card room based on the MRN of the selected participants and the data were collected from these records.

### Data collection procedure

A data extraction tool was prepared from the national treatment protocol for the management of SAM [22], SAM registration booklet, health management information system (HMIS) register, SAM multi-chart and by reviewing articles [16, 17, 24, 29, 30]. The data extraction format used consisted of socio-demographic data (age, sex, residence), anthropometric measurements (height, weight, MUAC, edema), co-morbidities, types of SAM (marasmus, kwashiorkor or marasmus-kwashiorkor), feeding phase and types of feeding (F75, F100, plumpy nut), frequency of feeding and amount per feed, immunization status, admission & discharge date, referral address as well as medication given and outcomes of the treatment. Six data collectors (BSc nurses) and two supervisors were recruited based on their experience in the SAM management process. Data collectors received one day training on the collection tool and were only deployed to collect data once the principal investigator was convinced of their competency. The primary investigator of the study and the supervisors critically followed the data collection process to minimize missing information and inconsistencies.

### Data processing and analysis

**Fig 1:**
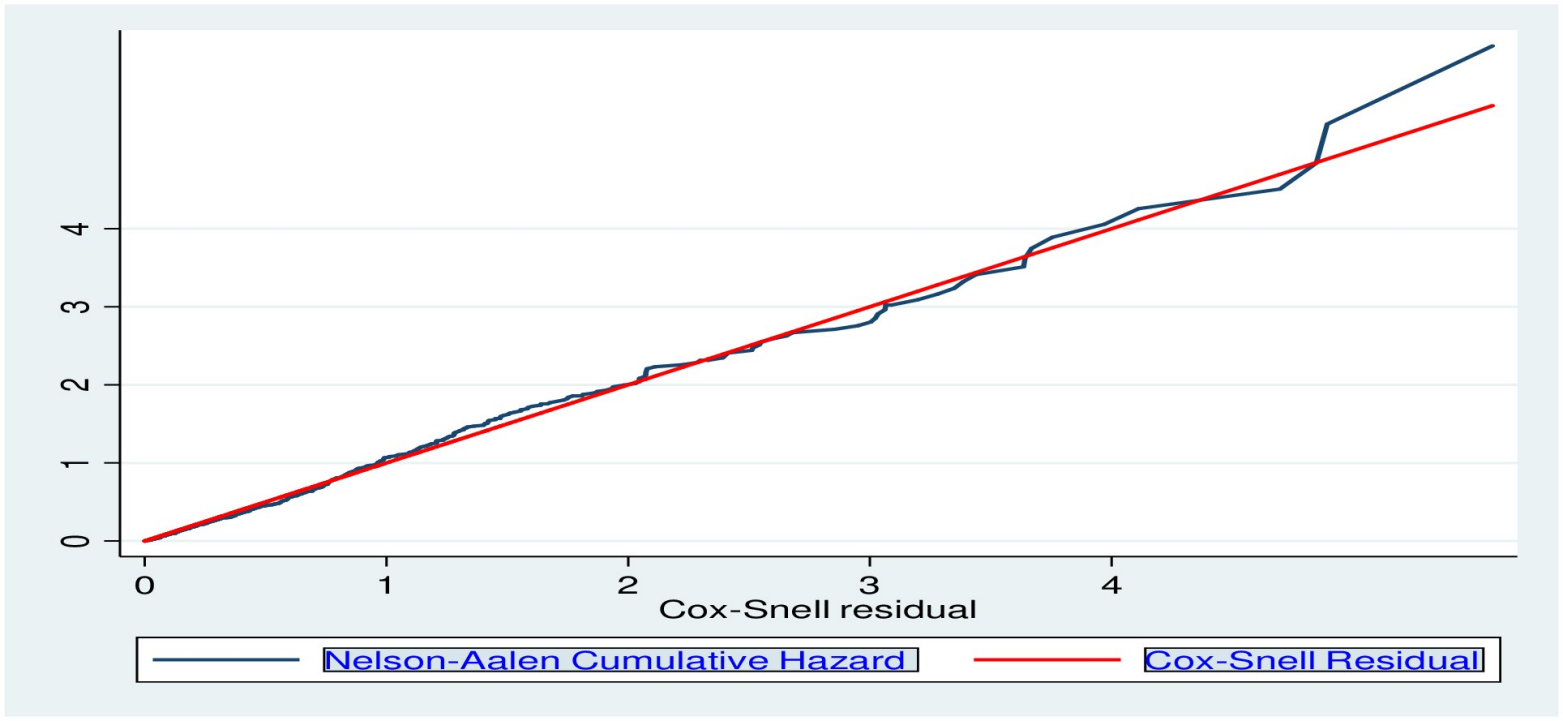
Model fitness by using Cox-Snail residual test to identify the incidence and predictors of time to recovery among SAM children at SPHMMC, Addis Ababa, Ethiopia, 2019.

Data was coded, entered, cleaned by Epi-info (version 7) software and exported to STATA version 15 (STATA Corporation, College Station Texas) software for analysis. The presence of missing values, possible outliers, and multicollinearity were checked through exploratory analysis. Kaplan Meier survival curve with the log-rank test was fitted to identify the presence of a difference in recovery rate among the categorical variables. Under-five children with SAM were followed in days from admission to the occurrence of the event (recovery). Person-time was calculated and the incidence was determined. In this study, person-time was reported in child-days. Child-days are total follow up times of each child from admission to the occurrence of the events (recovery or censored). Both bi-variable and multivariable Cox regression analyses were performed. Those variables with p≤0.25 in the bi-variable Cox-regression were selected for the multivariable Cox-regression analysis. All statistical tests were considered significant at 95% confidence interval. The final Cox regression model for fitness of the data and proportional hazards assumption was checked by the Schoenfeld residual test (the global test). The test revealed that the assumption was met with a p-value of the global test = 0.7. Unsteadiness of parameter estimate among variables in the final fitted model was checked by using variance inflation factor (VIF) and all the individual scores were less than 1.5 with the mean score of 1.16. The association was summarized by using adjusted hazard ratio and statistical significance was tested at 95% CI. Goodness of fit of the final model was checked using Nelson Aalen cumulative hazard function against Cox-Snell residual. The predict command was used to generate the Cox-Snell residuals from the model. The graph showed that the hazard function follows the 45-degree line very closely over time. This indicated that the final model was fit for the final model (Fig 1).

#### Ethics approval and consent to participate

Before the fieldwork, ethical clearance was obtained from St. Paul Hospital Millennium Medical College (SPHMMC) institutional review board (IRB). Patient cards were reviewed and informed consent was not required, since it was a retrospective document review. A formal letter of cooperation was submitted to the selected study area. Information obtained from the records was kept anonymous and confidentiality was maintained. Patient cards were coded and the data collectors knew each of the patient cards only by the codes given by the investigator.

## Results

### Socio-demographic and admission characteristics

Out of the 534 records, 515 (96.4%) were used for the final analysis. Nearly half of the study subjects were female and the majority (74%) were below the age of 24 months. 455 (88.3%) were newly admitted cases and around three quarters (76.5%) were non edematous. Regarding the feeding pattern, 306 (59.4%) children had a history of exclusive breastfeeding and 183 (35.5%) of children had a history of bottle feeding. Most, 370 (71.8%), of the study subjects resided out of Addis Ababa (Table 1).

**Table 1:** Socio-demographic and admission characteristics of children with SAM admitted in SPHMMC from 2012 to 2019, Addis Ababa, Ethiopia (n = 515).

Characteristics		Treatment Out Come		Total (%)
		Censored n (%)	Recovered n (%)	
Age	< 24 months	78 (20.5)	303 (79.5)	381 (100)
	≥24 months	32 (23.9)	102 (76.1)	134 (100)
Sex	Female	57 (22)	202 (78)	259 (100)
	Male	53 (20.7)	203 (79.3)	256 (100)
Admission	New Admissions	96 (21.1)	359 (78.9)	455 (100)
	Readmission	14 (23.3)	46 (76.7)	60 (100)
Residence	Addis	24 (16.6)	121(83.4)	145 (100)
	Out of Addis	86 (23.2)	284 (76.8)	370 (100)
Admission season	Wet season	57 (23.4)	187 (76.6)	244 (100)
	Dry season	53 (19.6)	218 (80.4)	271 (100)
Type of SAM	Edematous	33 (27.3)	88 (72.7)	121 (100)
	Non -Edematous	77 (19.5)	317 (80.5)	394 (100)
Appetite test	Passed	6 (20.7)	23 (79.3)	29 (100)
	Failed	38 (27.1)	102 (72.9)	140 (100)
	Unknown	66 (19.1)	280 (80.9)	346 (100)
Bottle feeding	Yes	47 (25.7)	136 (74.3)	183 (100)
	No	63 (19)	269 (81)	332 (100)
EBF	Yes	55 (18)	251 (82)	306 (100)
	No	55 (26.3)	154 (73.7)	209 (100)

EBF; Exclusive Breast Feeding.

### Treatments given to children

From the total of 515 study subjects, most (94%) were given IV antibiotics. The other commonly given treatments were F75 (85.4%), NG tube feeding (83.5%), F100 (81.9%), ReSoMal solution (57.7%) and Vitamin A (42.5%) (Table 2).

**Table 2:** Treatments given for under-five children with SAM admitted in SPHMMC from 2012 to 2019, Addis Ababa, Ethiopia (n = 515).

Variables		Treatment Out Come		Total N (%)
		Censored N (%)	Recovered N (%)	
IV antibiotics	Yes	102 (21.1)	382 (78.9)	484 (100)
	No	8 (25.8)	23 (74.2)	31 (100)
PO antibiotics	Yes	17 (11)	137 (89)	154 (100)
	No	93 (25.8)	268 (74.2)	361 (100)
IV fluids	Yes	49 (43)	65 (57)	114 (100)
	NO	61 (15.2)	340 (84.8)	401 (100)
Blood Transfusion	Yes	16 (32.7)	33 (67.3)	49 (100)
	NO	94 (20.2)	372 (79.8)	466 (100)
ResoMal Solution	Yes	66 (22.2)	231 (77.8)	297 (100)
	NO	44 (20.2)	174 (79.8)	218 (100)
F75	Yes	96 (21.8)	344 (78.2)	440 (100)
	No	14 (18.7)	61 (81.3)	75 (100)
F100	Yes	39 (9.2)	383 (90.8)	422 (100)
	No	71 (76.3)	22 (23.7)	93 (100)
Plumpy nut	Yes	5 (2.7)	181 (97.3)	186 (100)
	No	105 (31.9)	224 (68.1)	329 (100)
Vitamin A	Yes	27 (12.3)	192 (87.7)	219 (100)
	No	83 (28)	213 (72)	296 (100)
Folic Acid	Yes	49 (15.5)	268 (84.5)	317 (100)
	No	61 (30.8)	137 (69.2)	198 (100)
Zinc	Yes	19 (11.3)	149 (88.7)	168 (100)
	No	91 (26.2)	256 (73.8)	347 (100)
Iron	Yes	9 (5.8)	147 (94.2)	156 (100)
	No	101 (28.1)	258 (71.9)	359 (100)
Vitamin D	Yes	7 (15.6)	38 (84.4)	45 (100)
	No	103 (21.9)	367 (78.1)	470 (100)
De-worming	Yes	2 (2.1)	94 (97.9)	96 (100)
	No	108 (25.8)	311 (74.2)	419 (100)
NG tube feeding	Yes	86 (20)	344 (80)	430 (100)
	No	24 (28.2)	61 (71.8)	85 (100)

### Treatment outcomes and co-morbidities

**Fig 2:**
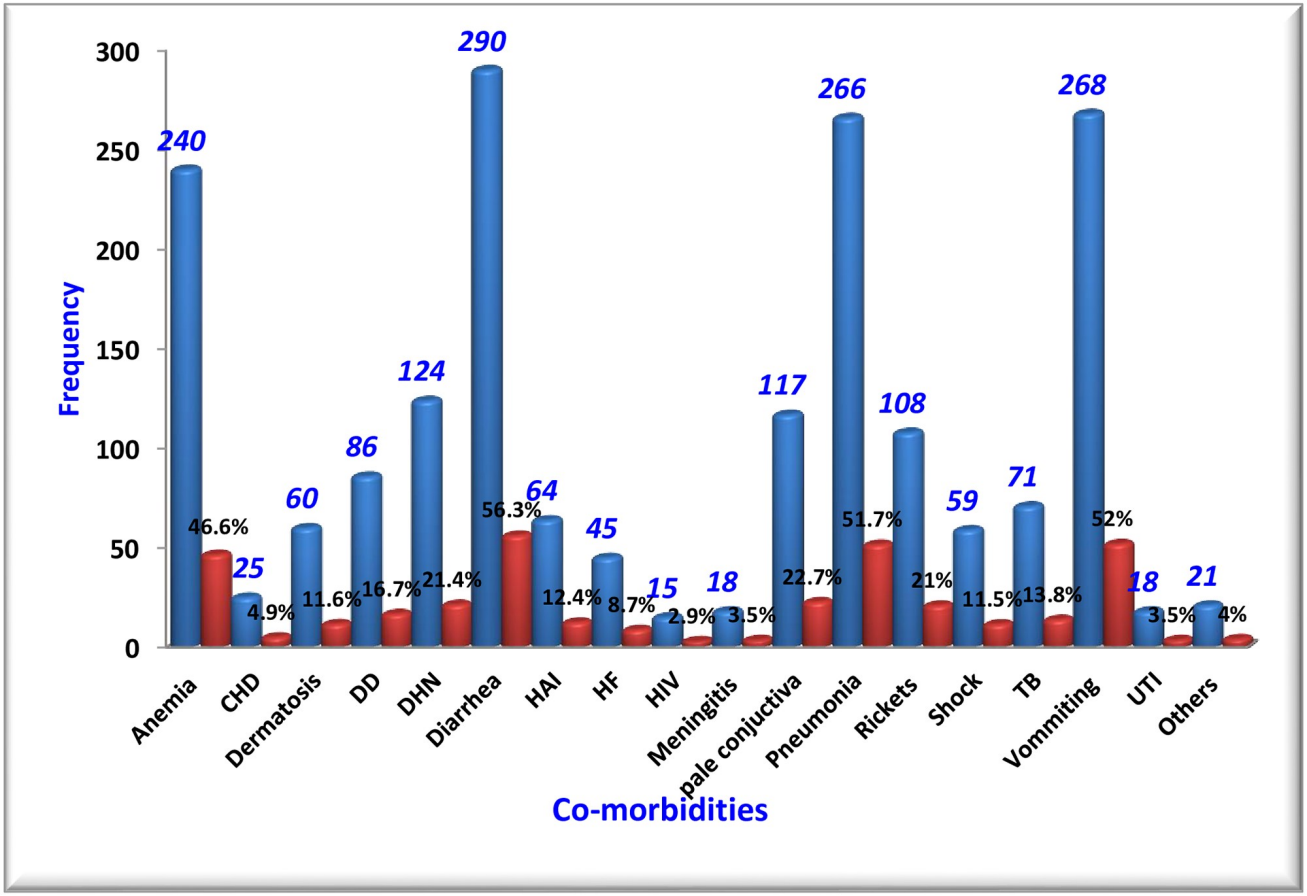
Co-morbidities of SAM children at SPHHMC from 2012 to 2019, Addis Ababa, Ethiopia, 2019. **TB**: Tuberculosis; **DHN**: Dehydration; **DD**: Developmental Delay; **UTI**; Urinary Tract Infection **HAI**: Hospital Acquired infections: **CHD**: Congenital heart Diseases; **HF**: Heart Failure.

In this study, the majority 407 (79%) of the study subjects recovered at the end of the follow up, but, 62 (12%), 46 (9%) ended in non-recovery and death, respectively. Diarrhea, vomiting, pneumonia, and anemia were the major co-morbidities of SAM in children with the proportion of 56.3%, 52%, 51.7%, and 46.6%, respectively. However, pulmonary hypertension (1.75%), pertussis (1.2%), pyloric stenosis (0.58%) and Guillain-Barre syndrome (0.58%) were the most infrequent co-morbidities found (Fig 2).

### Survival estimates for time to recovery

**Fig 3:**
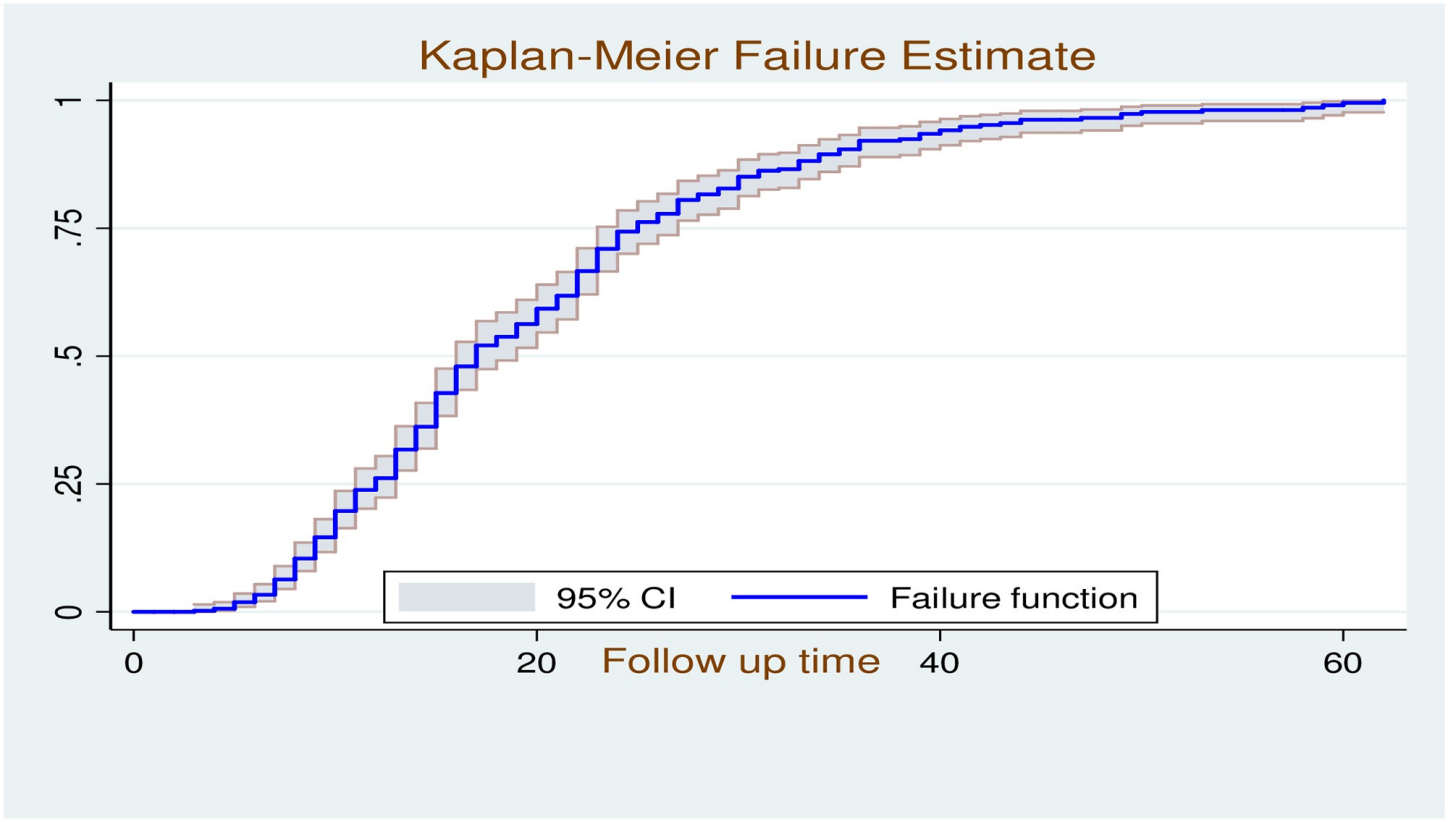
Log rank survival estimates for time to recovery among SAM children in SPHMMC form 2012 to 2019.

The over follow-up time was 8672 child -days with hospital incidence of cure rate of 46 per 1000 child-days (95% CI: 0.042, 0.051). The median duration of hospital stay was 15 days (IQR: 10, 23). The recovery rate in the 7^th^, 10^th^, 15^th^, and 30^th^ day of admissions were 8.9, 19.8, 30.9 and 43.5 per 1000 child-days, respectively (Fig 3). In this study the mean time of recovery was 20 (95% CI: 18.96, 21.14) days and the median time of recovery was 17 days (95% CI: 16, 19). Regarding the incidence density rate (IDR) of recovery, children who were given vitamin A had maximum recovery rate with the IDR of 52 (95% CI: 45.1–59.9) per 1000 child-days, whereas children who didn’t take F100 had the lowest recover rate with the IDR of 20.4 (95% CI: 13.3–31.3) per 1000 child-days (Table 3).

**Table 3:** Incidence density rate of recovery stratified by selected variables among severely malnourished under five children admitted to SC in SPHMMC, 2012–2019 (n = 515).

Variables		Frequency	Child-days	Recovered	RR (95% CI)
Over all	515		8672	399	46 (41.7–50.8)
TB	Yes	441	1624	46	28.3 (21.2–37.8)
	NO	71	7048	353	50 (45.1–55.6)
Anemia	Yes	240	4408	180	40.8 (35.3–47.3)
	NO	275	4264	219	51.4 (45–56.8)
Meningitis	Yes	18	428	11	25.7 (14.2–46.4)
	NO	497	8244	388	47.1 (42.6–51)
Shock	Yes	59	887	30	33.8 (23.6–48.4)
	No	456	7785	369	47.4 (42.8–52.5)
Fever	Yes	144	2681	109	40.7 (33.7–49.1)
	No	371	5991	290	48.4 (43.1–54.3)
LOC	Altered	35	617	19	30.8 (19.4–48.3)
	Normal	480	8055	380	47.2 (42.7–52.2)
Pale conjunctiva	Yes	117	2168	83	38.3 (30.9–47.5)
	No	398	6504	316	48.6 (43.5–54.2)
Vaccine status	Altered	331	5364	261	48.7 (43.1–55)
	Normal	184	3308	138	41.7 (35.3–49.3)
IV fluid infusion	Yes	114	1940	63	32.5 (25.4–41.6)
	No	401	6732	336	49.9 (44.8–55.5)
Blood Transfusion	Yes	49	1005	33	32.8 (23.3–46.2)
	No	466	7667	366	47.7 (43.1–52.9)
F 100	Yes	422	7643	378	49.5 (44.7–54.7)
	No	93	1029	21	20.4 (13.3–31.3)
Vitamin A	Yes	219	3636	189	52 (45.1–59.9)
	No	296	5036	210	41.7 (36.4–47.7)
Bottle Feeding	Yes	183	3239	136	42 (35.5–49.7)
	No	332	5433	263	48.4 (42.9–54.6)

### Predictors of time to recovery

**Fig 4:**
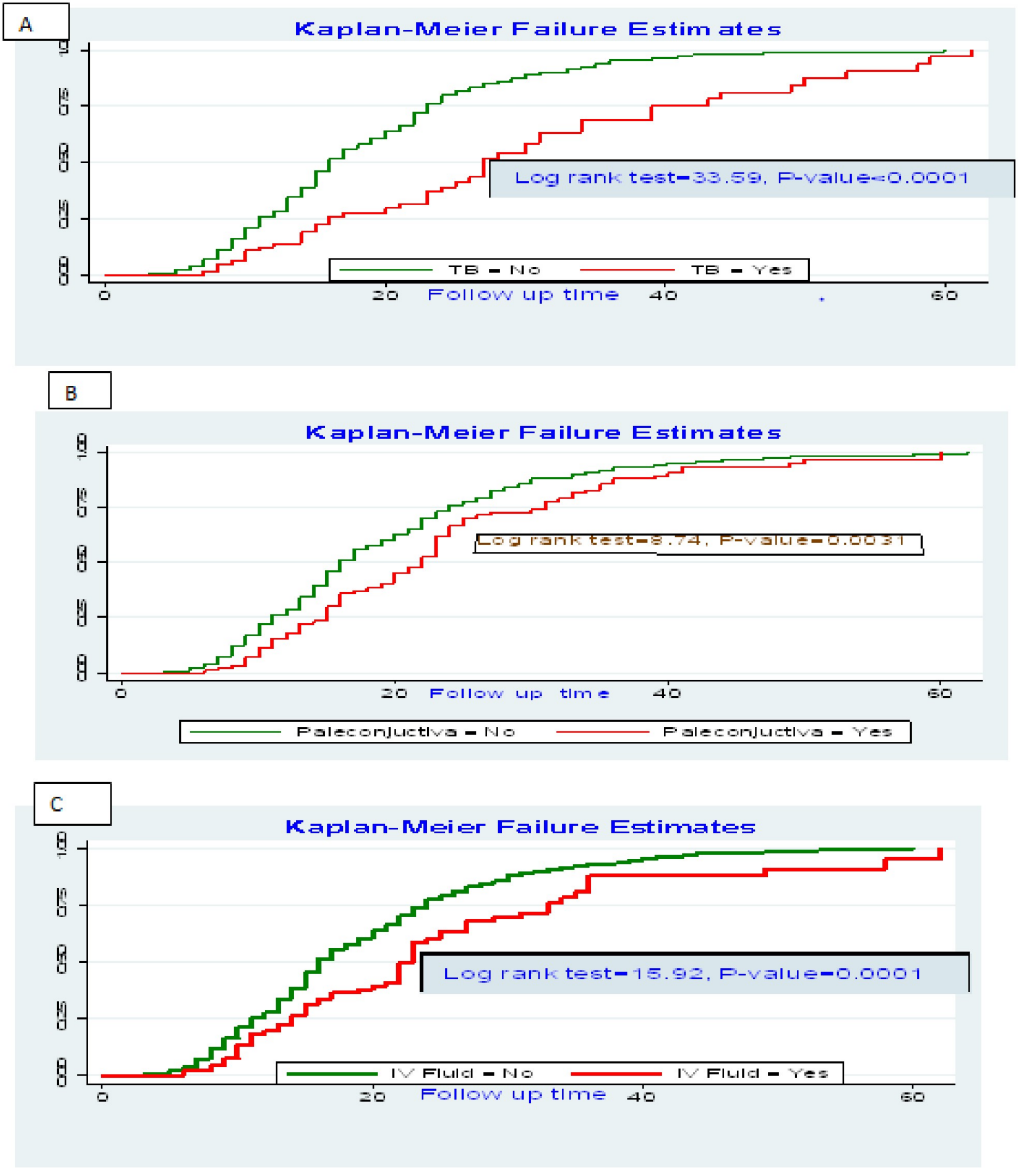
Log rank survival estimates for time to recovery among SAM children with independent predictors in SPHMMC, Addis Ababa, Ethiopia form 2012 to 2019 (A: Survival estimate with TB; B: Survival Estimate with pale conjunctivitis; C: Survival Estimate with IV fluid infusion).

**Fig 5:**
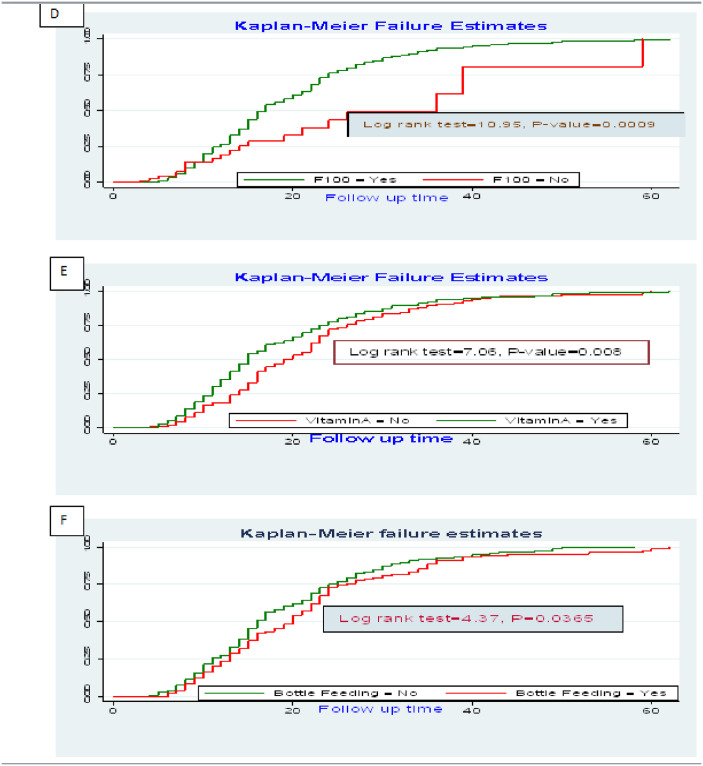
Log rank survival estimates for time to recovery among SAM children with independent predictors in SPHMMC, Addis Ababa, Ethiopia form 2012 to 2019 (D: Survival Estimate with F100; E: Survival Estimate with Vitamin A: F: Survival Estimate with bottle feeding).

In multivariable Cox regression analysis, TB, pale conjunctivitis, IV fluid infusion, F100, vitamin A supplementation and history of bottle feeding were found to be independent predictors of time to recovery from SAM. Children with no TB infection were 56% more likely to recover earlier than those who had TB (AHR, 0.44 & 95% CI: 0.32, 0.62). Children having pale conjunctiva on admission were 33% less likely to recover from SAM than their counterparts (AHR, 0.67 & 95% CI: 0.52, 0.88). Those children who were given IV fluids were 29% less likely to recover as compared to those who were not given IV fluids as part of the SAM treatment protocols (AHR, 0.71 & 95% CI: 0.51, 0.98). The therapeutic food named F100 was found to be significantly associated with recovery of children and children who were given F100 were 1.63 times more likely to recover from SAM than those who were not given F100 (AHR, 1.63 & 95% CI: 1.04,2.54). Children who took vitamin A as part of the SAM treatment were 1.3 times more likely to recover from SAM than their counterparts (AHR, 1.3& 95% CI:1.07, 1.59). The recovery rate of children who had history of bottle was 0.79 times than those who had a history bottle feeding (AHR, 0.79 & 95CI%: 0.64–0.98) (Table 4). The Kaplan Meier failure estimates of the independent predictors were also drawn with log-rank tests (Figs 4 and 5).

**Table 4:** Bi-variable and multivariable Cox regression model showing the distribution of factors associated with time to recovery in severely malnourished children admitted to SC of SPHMMC from 2012–2019 (N = 515).

Independent variables		Treatment outcome		CHR (95% CI)	AHR (95% CI)
		Censored N (%)	Recovered N (%)		
TB	Yes	25 (35.2)	46 (64.8)	0.41 (0.3–0.57)	0.44 (0.32–0.62)*
	NO	85 (19.1)	359 (80.9)	1	1
Anemia	Yes	58 (24.2)	182 (75.8)	0.7 (0.57–0.85)	0.85 (0.68–1.06)
	NO	52 (18.9)	223 (81.1)	1	1
Meningitis	Yes	7 (38.9)	11 (61.1)	0.42 (0.23–0.78)	0.72 (0.37–1.4)
	NO	103 (20.7)	394 (79.3)	1	1
Shock	Yes	28 (47.5)	31 (52.5)	0.66(0.45–0.96)	0.96 (0.61–1.5)
	No	82 (18)	374 (82)	1	1
Fever	Yes	33 (22.9)	111 (77.1)	0.75 (0.6–0.93)	0.89 (0.71–1.12)
	No	77 (20.8)	294 (79.2)	1	1
LOC	Altered	16 (45.7)	19 (54.3)	0.54 (0.33–0.87)	0.76 (0.46–1.26)
	Normal	94 (19.6)	386 (80.4)	1	1
Pale conjunctiva	Yes	34 (29.1)	83 (70.9)	0.7 (0.55–0.88)	0.67 (0.52–0.88)*
	No	76 (19.1)	322 (80.9)	1	1
Vaccine status	Altered	65 (19.6)	266 (80.4)	1.33 (1.07–1.64)	1.19 (0.96–1.45)
	Normal	45 (24.5)	139 (75.5)	1	1
IV fluid infusion	Yes	49 (43)	65 (57)	0.59 (0.45–0.77)	0.71 (0.51–0.98)*
	No	61 (15.2)	340 (84.8)	1	1
Blood Transfusion	Yes	16 (32.7)	33 (67.3)	0.53 (0.36–0.77)	0.81(0.53–1.22)
	No	94 (20.2)	372 (79.8)	1	1
F 100	Yes	39(9.2)	383(90.8)	2.02 (1.3–3.14)	1.63 (1.04–2.54)*
	No	71 (76.3)	22 (23.7)	1	1
Vitamin A	Yes	27 (12.3)	192 (87.7)	1.3 (1.06–1.57)	1.3 (1.07 (1.59)*
	No	83 (28)	213 (72)	1	1
Bottle Feeding	Yes	47 (25.7)	136 (74.3)	0.8 (0.65–0.99)	0.79 (0.64–0.98)*
	No	63 (19)	269 (81)	1	1

CHR: Crude hazard ratio; AHR: Adjusted hazard ratio; LOC: level of consciousness.

*, Independent predictors of time to recovery from SAM at p-value<0.05.

## Discussion

In this study, the recovery rate of under-five children from SAM in SPHMMC was determined from 2012 to 2019. A total of 515 SAM children were followed for 8672child-days making the incidence density rate of recovery 46 per 1000 child-days (95% CI: 0.042–0.0515) and the mean and median time of recoveries were 20 (95% CI: 18.96, 21.14) days and 17 (95% CI: 16, 19) days, respectively.

At the end of the follow-up, 79% of children recovered from SAM which is in line with the finding of a study conducted in southern Ethiopia [31]. The recovery rate in this study was relatively higher than the national SAM management standards and the sphere standards that recommended recovery rates of 75% under the current SAM management guidelines [22, 32]. The recovery rate in this study was also higher than the findings various studies performed in different locations of Ethiopia [12, 14, 16, 17, 20, 21, 24, 26, 27, 30, 33–40], of which ten [12, 14, 16, 17, 20, 26, 27, 33, 36, 40], five [24, 30, 34, 35, 39], two [37, 38], and one [21] were from Northern, Southern, Western and Easter Ethiopia, respectively. However, it was also lower than previous studies done in Ethiopia [13, 23, 25, 41], of which two of them were from Northern Ethiopia [23, 41] and others were from Western [13] and Southern [25] Ethiopia. These discrepancies could be attributed to differences in the organizational set-up, study and sample population, socio-demographic differences of the study subjects and variation in study settings.

In the current study, the overall incidence density rate of recovery was determined and it was 46 per 1000 child-days. This was relatively higher as compared to the previous findings conducted in Ethiopia, where the incidence recovery rates ranged from 22.7 per 1000 child-days % to 38 per 1000 child-days [16, 24, 25, 36]. The median time of recovery from SAM was 17 (95% CI: 16, 19) days. This is in-line with a study done by Fikrie et al. [24]. The median duration of recovery in this study was higher than the median duration of recovery of other Ethiopian studies [16, 17, 20, 26, 27, 36], but lower than some other studies done at the various stabilization centers in the country [25, 30, 31]. These variations could be due to differences in the quality of care, differences in the qualifications of care providers and in the organization of stabilization centers. The late detection of SAM and late referral to the stabilization centers might also contribute to the differences in the durations of recovery.

In this study, the presence of tuberculosis, pale conjunctiva IV fluid infusion and history of bottle feeding were found to be inversely related to time to recovery. In addition, vitamin A and F100 supplementation were found to enhance the time to recovery in children from SAM. Children having TB as co-morbidity along with SAM were less likely to recover earlier than those who did not have TB. This is in-line with the findings studies conducted in Southern Ethiopia [24] and North West Ethiopia [17], where TB was the main predictor affecting the overall incidence density rate of recovery. This is likely due to the fact that TB is an immunosuppressive disease and when combined with SAM, there is an increased risk of death [14, 42]. In the present study anemia was one of the commonest co-morbidities and pale conjunctiva (a common clinical feature of anemia) was found to be significant predictor inhibiting the recovery of children from SAM. This finding is in-line with other findings in Ethiopia that reported anemia as a main predictor affecting the recovery of children from SAM [14, 16, 17, 38]. Likewise, IV fluid infusion was found to decrease the recovery time of children from SAM by 29% and this was consistent with the finding of a study conducted in Southern Ethiopia [24]. Moreover, children who did not have a history of bottle feeding were 21% more likely to recover faster than children with a history of bottle feeding. This could be due to the fact that bottle feeding usually is associated with diarrheal diseases which could lead to poor recovery from SAM [43].

Similarly, the therapeutic food F100 and vitamin A were independent predictors enhancing the time of recovery from SAM. Children who took F100 were 1.63 times more likely to recover earlier as compared to their counterparts and this finding coincided with other findings in Ethiopia [12, 24]. F100 is crucial for appropriate weight gain in children and the effect is very significant for infants less than the age of six months [44]. The other possible explanation is that in this study 15.34% of study subjects were children below 6 months of age, and diluted F100 is given for these children from admission to discharge. The other reason could also be due to fact that censored cases (died, defaulted and transferred) might not take F100. Vitamin A supplementation as part of the management of SAM was found to decrease to the time of recovery of children. Children who were given vitamin A were 1.3 times more likely to recover faster than children who were not given Vitamin A. This was in-line with the finding of the study done in Seqota area, Ethiopia [14]. The possible explanation for this could be elucidated by the fact that Vitamin A is vital to boost the immune system and prevent diarrheal diseases. This prevents malnutrition and could indirectly shorten the time of recovery in children from severe acute malnutrition.

### Limitations and strengths of the study

The main strengths of this study include the use of the Cox proportional hazards assumptions and the model was fitted based on the predictor variables. However, the main limitation was difficulty in finding lost records from the card rooms, which might result in inaccurate information. The other limitation of this study was the inability to include the variables that were vague to read for the final analysis and this could lead to an inappropriate estimation of the predictors as well as the outcomes. Besides, inappropriateness of the anthropometric data prohibited us from following the prognosis of children based on anthropometric data. This study is a retrospective study and some vital statistics like HIV sero status might not be registered in patient cards. This could be the reasons for under reporting of sero status, though HIV testing is mandatory while children are admitted to hospital with SAM., which became a bottle neck for us to estimate the real effect of HIV/AIDS on the recovery rate of children with SAM.

## Conclusion

In conclusion, the recovery rate of children with SAM was relatively higher than the Sphere standard and the Ethiopian national standard. The presence of TB and pale conjunctivitis were the co-morbidities affecting the outcome of children from SAM. IV fluid infusion, F100 and vitamin A supplementation were also the treatment related factors determining the recovery rate of children with SAM. History of bottle feeding was also found to be the independent predictor of time to recovery of children. Co-morbidities need to be managed as early as possible and the treatments given during SAM management need to be given with precaution.

## Supporting information

S1 Data (XLSX)

## Abbreviations

AHR: adjusted hazard ratio
CHR: crude hazard ratio
CI: confidence interval
HMIS: Health Information Management system
HR: hazard ratio
IDR: incidence density rate
IV: Intravenous
LMICs: low and middle income countries
MRN: Medical Registration Number
MUAC: middle upper arm circumference
NNP-II: Second National Nutrition Program of Ethiopia
SAM: Severe acute malnutrition
SCs: Stabilization Centers
SPHMMC: St. Paul’s Hospital Millennium Medical College
SPSS: Statistical Package for Social Sciences
TB: Tuberculosis
VIF: variance inflation factor
WHO: World Health Organization
